# Classic motor chunking theory fails to account for behavioural diversity and speed in a complex naturalistic task

**DOI:** 10.1371/journal.pone.0218251

**Published:** 2019-06-13

**Authors:** Joseph J. Thompson, Caitlyn M. McColeman, Mark R. Blair, Andrew J. Henrey

**Affiliations:** 1 Department of Psychology, Simon Fraser University, Burnaby, Canada; 2 Cognitive Science Program, Simon Fraser University, Burnaby, Canada; 3 Department of Statistics and Actuarial Sciences, Simon Fraser University, Burnaby, Canada; Arizona State University & Santa Fe Institute, UNITED STATES

## Abstract

In tasks that demand rapid performance, actions must be executed as efficiently as possible. Theories of expert motor performance such as the motor chunking framework suggest that efficiency is supported by automatization, where many serial actions are automatized into smaller chunks, or groups of commonly co-occuring actions. We use the fast-paced, professional eSport StarCraft 2 as a test case of the explanatory power of the motor chunking framework and assess the importance of chunks in explaining expert performance. To do so, we test three predictions motivated by a simple motor chunking framework. (1) StarCraft 2 players should exhibit an increasing number of chunks with expertise. (2) The proportion of actions falling within a chunk should increase with skill. (3) Chunks should be faster than non-chunks containing the same atomic behaviours. Although our findings support the existence of chunks, they also highlight two problems for existing accounts of rapid motor execution and expert performance. First, while better players do use more chunks, the proportion of actions within a chunks is stable across expertise and expert sequences are generally more varied (the diversity problem). Secondly, chunks, which are supposed to enjoy the most extreme automatization, appear to save little or no time overall (the time savings problem). Instead, the most parsimonious description of our latency analysis is that players become faster overall regardless of chunking.

## Introduction

Performance timings of motor behaviour are suggestive of higher level processes that control entire sequences (i.e., ‘chunks’, e.g., [1–3]). These chunks have often be used as an explanation for performance improvements during learning. For example learning curves have been explained in terms of the acquisition of these chunks by several researchers [4–7]. Critically, chunked sequences are advantageous for performance because chunks are executed quickly [8], and because automatization frees up cognitive resources for higher-level processing [9, 10].

Our understanding of motor chunks comes from a number of laboratory-based tasks, such as the discrete sequence production task [11], in which participants are taught sequences, and the timings of constituent actions are then explored. While much research has been done on motor chunking in the laboratory, little has been done outside it and so the relationship between learning and chunking in more naturalistic tasks is unclear. Further, in most laboratory situations, participants’ goals are entirely structured around producing the small number of sequences quickly, and there is little else for them to do. To what extent chunking is related to learning in more complex tasks where sequences might not repeat as often, or be the exclusive focus of the participants is also unknown. Most of the recent evidence on chunking and hierarchical control in natural performance comes from typing [8, 12–14]. As a task, typing is a natural one to study as it is prevalent outside the lab, and performance timings can be easily studied. Because words are naturally thought of as chunked sequences of letters, however, the importance of chunks is possibly exaggerated in these domains. Additional study of chunking in naturalistic contexts is therefore warranted.

Importantly, the Big Data age has afforded cognitive science new avenues for evaluating psychological theory [15]. Video games, in particular, have shown to be a useful domain for examining learning outside of the laboratory [16]. Digital archives left behind by thousands of gamers of varying skill has allowed researchers to quantify the extent to which expert-novice comparisons could prove misleading [17] and to identify age-related changes predicted by theory [18]. More recent work has even begun to use such datasets to examine motor-chunks [19], highly practiced sets of actions that a person can execute with the same ease that they can execute individual actions. However, naturalistic task environments are complex, and this has compelled the prior research to make strong assumptions in their detection of motor chunks, such as the assumption that chunks could be indexed by attentional shifts. The present work takes a more theoretically neutral approach, and utilizes a new chunk detection method to test see how the diversity of motor sequences, and their latencies, change with expertise.

The goal of the present study is to investigate chunking in a naturalistic and complex cognitive/motor task. We document the prevalence of chunks in such a domain, StarCraft 2, and their impact on performance timings as expertise increases. Our approach was to generate predictions from a simple account based on broadly supported principles, and ask, to what extent is a simple account of motor chunking sufficient to explain expert performance timings. The extent to which, and then manner in which, this account fails will be instructive for more complex theories of motor chunking and performance timings in sequence learning. Finally, regardless of the implication for particular theories, our research expands the set of naturalistic domains in which motor chunking has been studied, providing important descriptive data.

To achieve our research goals we need meet three requirements. First, we need a task domain in which sequences of actions can be recorded with precise timings, and one in which chunks might be naturally relevant to, and beneficial for performance. Second, we need to develop a set of predictions from existing work on chunking. Finally, to enable us to test those predictions, we need a method of detecting chunks in a stream of natural responses. In the present work we evaluate action sequences using the digital records of real time strategy video game StarCraft 2 and thus investigate a domain in which chunks are not the focus of the task itself.

### The utility of StarCraft 2 as a domain for studying chunking

StarCraft is a real-time strategy game. It is a strategic game like chess, except that players are not required to take turns, do not have to wait for their opponent to move, and are not given complete information about an opponent's units. In this kind of game, making moves more rapidly and efficiently than one’s opponent is a huge advantage. Player screens view only one selected portion of the board or map in detail at any time, but have access to gross information on a miniature map in the corner of their screens. The goal of the game is to collect resources, produce an army, and defeat the opponent’s army. Basic mechanics of the game involve using the keyboard and mouse to (a) select units or structures and (b) direct their movements and activities in order to further player goals.

One of the most appealing incentives to study games like StarCraft 2 is methodological. It promises passive, non-invasive, and ethical data collection. In StarCraft 2, every action that impacts the game is automatically collected during play in a timestamped list. Players of all skill levels thus leave behind a digital trace of their performance by simply participating in their domain of excellence. Furthermore, eSports such as StarCraft 2 contains professional players with full time careers, who are sponsored by major corporations and compete for significant prize purses. These ambient telemetry data can bridge the laboratory and the real world in unprecedented ways.

StarCraft 2 is a near perfect domain in which to study motor chunking. To start, rapid execution is paramount and the drawbacks of automatic performance (e.g., [20]) are few. Much of this game, where the goal is to destroy a human opponent’s army through the management and command of one’s own resources, revolves around the game mechanics of managing a civilization’s economy, producing an army, and overseeing the movements of game units. StarCraft 2 should greatly favour the use of highly automatized sequences of behaviour. Prior work has shown that the game favours speed greatly [17, 18]. This makes sense as faster players are able to issue more strategic commands (resulting in a more efficient economy and better positioned army). Relying on motor chunks in StarCraft 2 play provides important speed benefits at little strategic cost. Further, games of StarCraft 2 produce a timestamped record of actions each player provides, and so provides the measurements of the timings of actions that are equivalent to data from the laboratory. Finally, StarCraft 2 is a domain the authors of the present work know very well. Dr. Henrey was a master league player, and all other authors were gold league or better. In the course of our previous research [17, 18, 19] we have interacted with players of all levels in the StarCraft 2 community and discussed expert play with professional players. This expertise allows us to temper our interpretations of data with deep domain knowledge, and also temper our domain intuitions with a deep familiarity with the relevant data. By using StarCraft as a domain, we thus avoid some of the pitfalls of interpreting big data in domains of only moderate familiarity.

### General predictions from the Motor Chunking Framework about chunk use and acquisition

The second requirement for our study is that we generate some expectations about what should happen to actions timings as a result of the accumulation of motor chunks. One problem is while there are many commonalities amongst the theoretical ideas surrounding chunking, there are also many differences. For example, classic theories of automaticity allow for individuals to switch to deliberate processing [5], and recent applications of motor chunking theories to typing performance allow for interruptions even at the highly automatized level of the keystroke [14]. Other frameworks allow for a variety of speedy motor-execution strategies, making it problematic to think of chunks as the unitary sources of expert speed [21]. While such accounts may indeed make predictions about actual performance outside of the laboratory, the present state of research in StarCraft 2 is at too early a stage for us to make suitably specific predictions. For example, Verwey and colleagues [21] argue that motor-representations (chunks), perceptual-representations, and even verbal-representations can potentially give rise to speedy performance. However, there is currently no method for parsing out whether a behaviour occurring in natural StarCraft 2 performance was brought about by a motor or spatial representations, or whether a particular speed gain is primarily due to changes in the motor system. *Chunks*, *for the purposes of this investigation*, *are automatized sequences (i*.*e*., *motor-chunks) of commonly used actions in a task*. This definition has broader relevance than prior definitions of ‘chunk’ in StarCraft 2, where chunks were just behavioural sequences falling between shifts of attention [19]. The broad definition fits with a broader reading of the literature on reaction time [8], neuropsychology [22, 23], and computational modelling [24]. We derive a simple theory of chunking from this literature which we call the motor-chunking framework (MCF).

**Fig 1:**
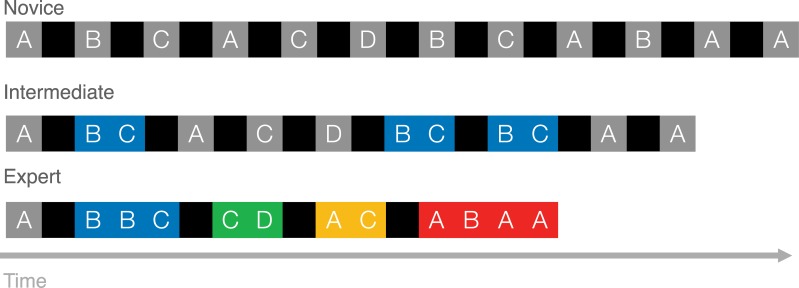
The motor chunking framework (mcf) description of expert performance as chunk accumulation. In the novice, actions are preceded by cognitive-motor planning. As action sequences are unitized into chunks, planning is done at the beginning of sequences only, thus saving time on subsequent actions and producing the well-established timing pattern within chunks of slower first actions and faster subsequent actions. As expertise accumulates, chunks increase in size and number, taking up a larger and larger proportion of the actions produced, further speeding performance.

It is worthwhile seeing how the MCF would construe the skill development of a hypothetical player. To play StarCraft 2 well, players must habitually cycle through actions pertaining to building their economy, training their army, and positioning their army. Initially, the behavioural sequences associated with basic game functions, such as training a basic fighting unit, should be variable (see Novices from Fig 1). This level of skill would be akin to the 'hunt and peck' method of novice typists, where each keystroke involves a search [8]. Our hypothetical player remembers doing each game operation in a variety of ways so, when its time to train a fighter, a ‘race’ between these memories might decide which sequence can be most speedily executed [5]. However, after some experimentation with various methods for training fighting units (e.g. by using the mouse or some combination of mouse and keyboard), it becomes clear that the most efficient is to assign their production structures to a number key (HotKey) so that these structures can be selected with a single keystroke (HotKeySelect), followed by a second keystroke which initiates the structure to train a unit.

Of course, even these ‘HotkeySelect-Train’ keyboard sequences are sluggish at first. The player must recall which number key selects relevant production structures to which keys will order the structures to train a unit. But this sequence should eventually be chunked and automatized (See the ‘BC’ sequence of intermediate players depicted in in Fig 1; [9, 10]. On one specific account, one process (the 'outer loop’) need only decide to train a fighting unit, and an independent processes (the ‘inner loop’) will take care of the required keystrokes [12]. Most importantly, the MCF predicts that all the basic game functions of StarCraft 2 (which mostly involves training units, building economic and military structures, commanding the army to move, and commanding the army to attack) will eventually be dominated by a relatively small number of specific behavioural sequences (see expert skill level, Fig 1). This is because every execution of an already efficient behavioural sequence further contributes to its automatization, purchasing an even larger speed advantage in future races. Thus the StarCraft 2 player, according to the MCF, uses chunking to greedily save time whenever possible, replacing slower and more deliberate sequences with automatized chunks.

We begin from the notion that the MCF will play a major role in the explanation of skilled StarCraft 2 performance. This directly leads to three predictions that might be harder to deduce from more nuanced chunking theories 21A e.g., 19]. The first two predictions are relevant to the diversity of motor sequences while the third prediction speaks to performance latencies. According to our first prediction, with expertise, StarCraft 2 players should exhibit an increasing number of chunks (See Fig 1) because these are a source of speed, consistency, and accuracy.

The second prediction of the MCF is that chunks will replace more clumsy and variable attempts at performing basic actions. That is, the proportion of actions that fall within chunks should increase with skill (See Fig 1). Prediction 2 follows from the MCF because, for any given game mechanic, such as training a unit, a player should be able to more efficiently accomplish this action by replacing deliberately controlled behavioural sequences with chunks.

The third prediction from our application of the MCF to StarCraft 2 is that chunks save players a lot of time. There are three reasons why. First, the automatized sequences in basic laboratory typing tasks are faster [8], and this is true both for the relatively sluggish first actions of keystroke sequences, which are thought to reflect costs emerging from multiple levels of control [12], and for the latencies following the first action (in keyboarding tasks this is usually called the inter-keystroke interval, [14], which reflect processing of the 'inner loop’ except in cases of interruption [13]. Secondly, this automatization is thought to free up cognitive resources for higher-level planning [9, 10]. In a complex task this could save time by alleviating the need to plan under dual-task constraints. Finally, it’s expected that players undergo some exploration into the possible ways of performing StarCraft 2 functions. In a genuine domain of expertise such as StarCraft 2 (which is played professionally and for which there is dedicated training), it’s expected that players are trying to improve [25], and will therefore intentionally avoid automatizing highly inefficient methods for performing basic game functions. The chunks chosen by experts, therefore, are also likely to be better methods for completing the task.

### Finding chunks in StarCraft 2 data

The final requirements of our study is to be able to identify chunks in the actions and timings of StarCraft 2 players. While our specific method of detecting chunks is described in full in the Method section, it is worthwhile to give the reader a general idea of how this is to be done. There are several existing current chunk detection algorithms (e.g., [26, 27]) that classify chunks based on the structure of performance timings, and therefore already assume that expert speed of performance is derived from rapidly performed sequences. Given that we are dealing with a complex, naturalistic task, we want instead to be open to the possibility that such speeds are due to other sources, such as the reduction in cognitive load as other theories would predict. We therefore construct our chunk-detection algorithm around the assumption that experience with a motor sequence (i.e., prevalence) is a primary source of automaticity and chunk acquisition [5]. This will allow us to independently test our algorithm against data on performance timings and to ask questions about performance latencies. Our most important research questions will also be addressed using converging analyses that *do not* rest on this algorithm.

## 2. Methods

### Participants

3,330 StarCraft 2 players submitted a usable replay file of a one-on-one StarCraft 2 game. Participants also filled out surveys providing demographic information and character codes. The latter was used to verify player league (coded as 1–7 from least to most skilled in our dataset). 55 additional professional replays (coded as 8 in our dataset), were acquired from publicly accessible replay hosting websites. Initially replay files are in an unreadable proprietary format. Replays were then parsed with the SC2Gears [28] software into a series of timestamped actions and uploaded into a series of MySQL tables. Importantly, our conclusions are only expected to generalize to men, as only 0.9% (29 individuals) of the sample were women. The mean age of the sample was 21.6 (sd = 4.2). Both the present study and the collection of the raw digital archives received ethics approval through the Office of Research Ethics at Simon Fraser University (2011s0302), and all participants gave informed consent and digital game records through an online survey. The entire procedure was performed in accordance with the ethical standards laid down in Canada’s Tri-Council Policy Statement.

### Task

StarCraft 2 is methodologically ideal for several reasons. First, this method allows for fine grained measures of performance. Second, StarCraft 2 is a domain where rapid behaviour, and presumably chunking, is extremely adaptive. Third, Starcraft 2 is a domain that supports genuine experts with full-time practice regimes, and so data can reflect learning across thousands of hours of practice. Finally, while StarCraft 2 is like typing in emphasizing efficient performance, it is quite different as well. Behavioural sequences in StarCraft 2 are not organized into words, as in typing, which means that StarCraft 2 may not exaggerate the role of chunking. Furthermore, StarCraft 2 performance differs from typing in that it requires strategic thinking and management of dual-task demands [19].

### Action typology

The three predictions we wanted to test required an algorithm to identify chunks in StarCraft 2. In StarCraft 2 replay data, we extract a game record comprising all actions the players took and the time at which they were logged by the game engine. These data are classified in such a way as to allow the StarCraft 2 game engine to replay the game exactly. Importantly, this classification has face validity insofar as it will be familiar to StarCraft 2 players as actions they chose to take, such as constructing a building at a particular location, or selecting a particular unit. In total, we classify players’ actions into seven types:

1. Select: Players use the mouse to select units they wish to control.
2. Hotkey Select/Assign: Players can assign a set of units to a keyboard number (0:9), or select a set of units using a previously assigned hotkey.
3. Train: Players command a structure to train another unit. This is how players acquire armies that can be used to wage war.
4. Build: Players command a unit to build a new structure. These structures are the basis of the players civilization, and are required for building an army.
5. Right Click: A context-sensitive key. Can order units to move, attack a right-clicked target, or collect resources.
6. Ability: A collection of less frequently used commands and special abilities, most of which are restricted to one of the games unit types. See the supplementary materials of Thompson, Blair, Chen, & Henrey [17] for a complete list of StarCraft abilities.
7. Screen Shift: Screen shifts represent attentional shifts. These actions were derived from raw screen movement data as, much like gaze data, these are too numerous and chaotic for the present analysis. Instead, we follow Thompson, Blair, Chen, & Henrey [17], and aggregate screen movements into screen fixations using a Salvucci & Goldberg [29] algorithm. We then treat a movement of the screen as a distinct action type for the purposes of our analysis.

A player might have data a sequence like this: {Screen shift; select; train; hotkey select; right click; right click; right click; ability}. The dataset that results is a timestamped list of actions types in order of their initiation. This list is then processed to look for action sequences that might be chunks due to their prevalence.

### Prevalence-based chunk detection method

We designed an algorithm to extract chunks from veridical records of gameplay (see Thompson, Blair, Chen, & Henrey [17] for additional information about the digital archives used) from 3,385 StarCraft 2 game files, yielding 3,020 games indicated as containing potential chunks and a final set of 802,853 chunked actions.

One source of complexity in identifying probable chunks by looking at sequence frequency was that the base-rates of the 7 possible atomic actions are not equal in StarCraft 2 play, and so some sequences might be frequent simply because their atomic components are more common. To address this, we considered base rates of the actions making up sequences. A sequence was considered a chunk if it is more common than the base rates of the action types in the sequence would suggest. That is, we asked whether sequences are occurring more often than they should "by chance”. We borrowed a procedure to address the problem of uneven base rates from the literature on machine learning and text mining [30]. We began by considering each of the 7 action types as one of 7 actions (“words”), and we found the corresponding frequency of each word within the game. We divide these counts by the total number of actions (we call this *a*) to get 7 marginal proportions (pAttack, pBuild, and so on), one for each action type. We will considered as the null distribution the case where the player picks a word at random *a* times to construct the text (or in our case, the set of actions that constitute that a specific StarCraft 2 game).

We tested, for bigrams, trigrams, and four-grams, what the probability of attaining each n-gram K times is (under our base-rate corrected null distribution). To avoid excessive multiple testing, we limit ourselves to looking at a small selection that are motivated by the data itself. We use the technique of data splitting [30]: we develop a list of viable sequences to test from half of the data, and then test that list against the remaining half to ensure we don't bias the results.

The identification process follows the following steps:

1. The first step in the algorithm is to create a test set and a train set of data, which we accomplish by dividing the game into non-overlapping bigrams (e.g. the sequence 'XYXY' is divided into the two bigrams 'XY' and ‘XY’). Bigrams containing repeated 'Right Clicks', 'Trains', and 'Hotkeys' are marked and dropped from the analysis. In StarCraft 2 data, these actions are often found in long, uninterrupted, strings. These strings are potentially artifactual in the case of 'Trains' (holding down a single key can produce multiple train commands the latency of which does not depend on the player) or unrepresentative of meaningful Starcraft 2 behaviour in the case of 'Right Clicks' and 'Hotkeys' (both repeated right clicks at the same location and repeated selecting of different unit groups have no impact on the game but introduce abnormally low latencies).
2. We then distribute half the bigrams into a training and half into a test set, with n observations each. A game of 1,000 actions will therefore have 500 bigrams in total and 250 bigrams in its training set.
3. In the training set, we then compute the p-value for the bigram. Suppose we observe a particular bigram K times. Under the null distribution described above, the p-value is probability of observing this bigram at least K times if the player picked bigrams at random. Since the training set has a fixed number of bigrams, the null distribution of the number of occurrences of our bigram has a binomial distribution. The parameters of our binomial null distribution are n = n and p = p_i_p_j_, where pi and pj are the marginal proportions of the first and second actions in the bigram.
4. We order the p-values from the test set and then take the bigrams with the lowest p-values to the next stage with a limit of five. Since we have selected these bigrams through an optimization procedure, we expect that these estimates of the p-values will be optimistic. Therefore we discard the p-values generated from the training set and only move five bigrams forward to the next step.
5. We compute a new p-value for each bigram identified in the training set using only the test data and following the same approach above: determining the probability of observing each bigram K times. Since this test set is uncontaminated by the optimization done in the training set, these p-values are reliable.
6. Any bigram which has a p-value of < 0.05 in the test set is considered to be a chunk. That is to say, we believe the player was unlikely to generate this sequence at random, and thus conclude that it is an important sequence for that player.
7. We then repeat steps 1–7 using trigrams and four-grams.
8. Once we have a list of bigram chunks, we pass through the entire dataset, marking which actions fall within a chunk. Actions falling within chunks, but which are preceded by actions outside of a chunk, are labelled First Actions.

After the procedure is complete, we may have found up to 15 chunks (at most 5 each of length two, three and four sequences). One limitation of any chunk-detection method is that we may miss chunks. However, allowing for more sequences would lead to increased multiple testing, which is also undesirable. We can therefore think of the maximum number of chunks as a tuning parameter which reflects the tradeoff between trying to find true positives and eliminate false positives.

There is reason to think that allowing a maximum of 15 chunks per game will provide a reasonable balance of type I and type II error rates. First, each game contains hundreds, often thousands, of actions which guarantees a large training and testing set. This allows us respectable power. Secondly, we selected a parameter setting such that the chunks highlighted made sense given our knowledge of the game. This ensures that the chunks identified would make sense to a competent StarCraft 2 player. Third, only about 3% of the games analyzed reached the maximum of 15 chunks, suggesting that larger parameter settings would not result in many more chunks. Fourthly, our per-test alpha of 0.05, while less strict than using a Bonferroni family-wise correction, is stricter than Linkletter et al. [31], who argued that per-test alphas of 0.1 are reasonable in the context of exploratory work.

Our decision to limit our analysis to sequences of length 2, 3, and 4 was largely based on theoretical grounds. The primary methodological pursuit in this study is to describe the common behaviours in the game as they are experienced—and exhibited—by players. Through observation of player performance, and through the authors’ own firsthand experiences in the game, we recognize the importance of various action pairs (and trigrams, and four-grams) as having particular value in executing sub-goals in StarCraft 2. A common method for creating a building, for example, requires the player to select an appropriate worker, use the mouse to select the ‘build’ command, and use the mouse to select the desired type of building, and finally use the mouse to specify a location for the building. We hypothesized that expert players would achieve their impressive speeds by cycling through a small number of such chunks.

### Analysis strategy & key variables

Two of the key measures for the present study (the number of chunks, and the proportion of actions falling within a critical sequence) directly follow from the identification of chunks. The third key measure is *time savings*, which is derived by comparing action latencies of actions within chunks to the latencies of actions that are not-chunked. That is, the time savings for every action falling under a chunk is the difference between its actual latency and the mean latency to perform non-chunked actions of the same type. Importantly, we did not hypothesize that skilled players would have higher per-action time savings, as we expect that the automatized novice and intermediate levels would also have chunks which reach asymptotic speeds. Instead, we hypothesized that experts would have greater overall time savings because a larger proportion of their actions would be chunked. We therefore find it more useful to report a game's overall time savings (i.e., the sum of the per-action time savings).

Importantly, our work contains both hypothesis driven and exploratory analyses. On the one hand, our method of chunk-detection is intended to be used in a hypothesis driven manner, with careful error control in mind. However, the complexity of the task domain means that results will need to be interpreted carefully and many followup analyses will be necessary. For example, we began with planned analyses that speak to our chunk-detectors validity and then proceeded with planned analyses of the MCF’s three predictions. The predictions were tested with a series of 8x6 ANOVAs with factors of League (eight levels) and StarCraft 2 Species (six levels). However, where the MCF’s predictions were not observed we performed followup analyses to clarify the nature of this predictive failure. A primary purpose of these followup analyses was to establish whether our results were an artifact of the chunk-detector.

## 3. Results

**Fig 2:**
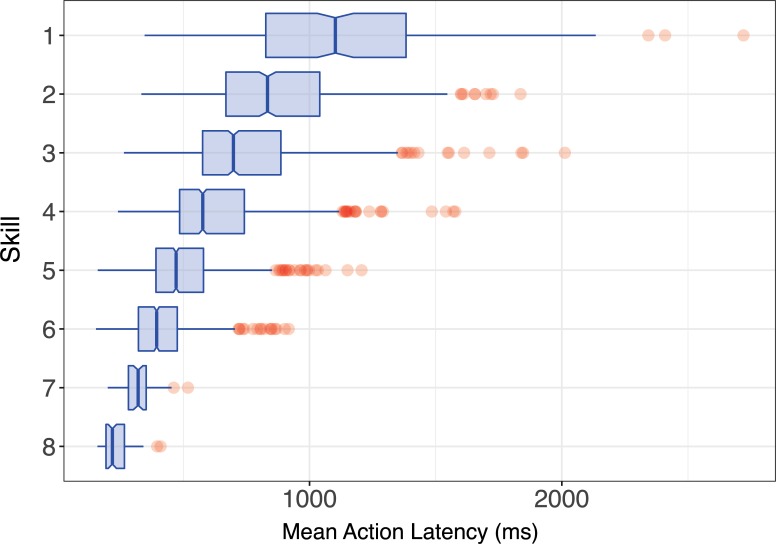
Mean action latency by skill level. Mean action latency by skill level, where 1 is the least skilled. This pattern of speeded expert performance is one of the empirical phenomena the MCF is attempting to explain.

We began by ensuring that our data contain expertise related changes in performance speeds. Fig 2 shows decreases in average time taken to complete an action for each of the 8 skill levels. The differences between skill levels that are clear in the figure were confirmed by statistical analysis. The nuisance factor ‘player species’ is added to our ANOVA models as StarCraft 2 players must select their species prior to play, and this choice impacts some game mechanics and, ultimately, a player's average action latency (F(5,3178) = 126.65; p<2e-16; η_p_^2^ = 0.17; for additional details, see S1 Text). Most importantly, there was a significant main effect of league (F(7,3178) = 421.49; p< 2.2e-16; η_p_^2^ = 0.481) on action latency, confirming that speed does indeed change with expertise (also see Fig 2).

The present study asks, how much of performance improvements can be attributed to the simple motor chunking account provided by the MCF. The decrease of action latencies in Fig 2 is thus the target of this explanatory account. Given these numbers, we can be more specific about what the MCF would predict regarding the prevalence and time-savings of chunks in StarCraft 2, if it were a complete account of performance improvements. Participants in our lowest skill level perform an action in an average of 1019ms, and participants in our highest skill level take only 234ms on average to perform an action. A theory of chunking thus has 785ms per action, and roughly 100ms of improvement per skill level to address. This is enough data to allow us to get a rough sense of what a chunking explanation of player speed might look like. If we estimate that chunks form about 10% of the actions at skill level 1, and about 80% of the actions at skill level 8, it would mean that a non-chunked action should take an average of 1100ms, and a chunked one only 100ms (thus chunking saves 1000ms per action). On this picture, every skill level increases the percentage of chunks by 10%, and thus decreases the average latency by 100ms. This gives us numbers which are roughly similar to those in Fig 2. To the extent that a chunked action saves less than 1000ms, the MCF account will need to posit a higher proportion of chunked actions to compensate. To the extent that chunks are less prevalent (less than 80% at skill level 8), they each need save even more time. Of course there may be other factors than chunking involved in the speedup of actions, and so we might find that chunked actions are only 500ms, not 1000ms, faster than non-chunked actions; conversely, chunks might comprise only 40%, not 80%, of the actions of experts. In those cases we could say that chunking accounts for roughly half of the obtained performance gains, and thus provide some assessment of what additional factors, or more complex accounts motor chunking.

After running our chunk-detection algorithm, only 365 players failed to have even one chunk. For the rest of our discussion, we restrict our analysis to the 3,191 participants who had actions that were at least nominated as potential chunks in the training set. Furthermore, most players had a unique set of common sequences. Of the 587 unique chunks, the most common was the bigram ‘Screen-shift—Select', which was critical in 56% of the games. Of the unique chunks identified, most were only identified in a handful of games (median = 4), though this distribution was quite skewed ($\bar{x}$ = 27.9; SD = 100.3).

To ensure that identified chunks exhibited established timing patterns, we defined ‘first actions’ as the first action in a sequence of chunked actions. The first actions of chunks are slower ($\bar{x}$ = 720 ms; SD = 377) than inter-action latencies ($\bar{x}$ = 563 ms; SD = 273; t(3019) = -32.733; p<2e-16; 95% CI = 147.1 : 165.84 ms). The chunks we identified solely by the *prevalence* of the sequence (above chance) do bear the established *timing* patterns of chunks [2, 3].

### Chunk prevalence and chunk diversity

**Fig 3:**
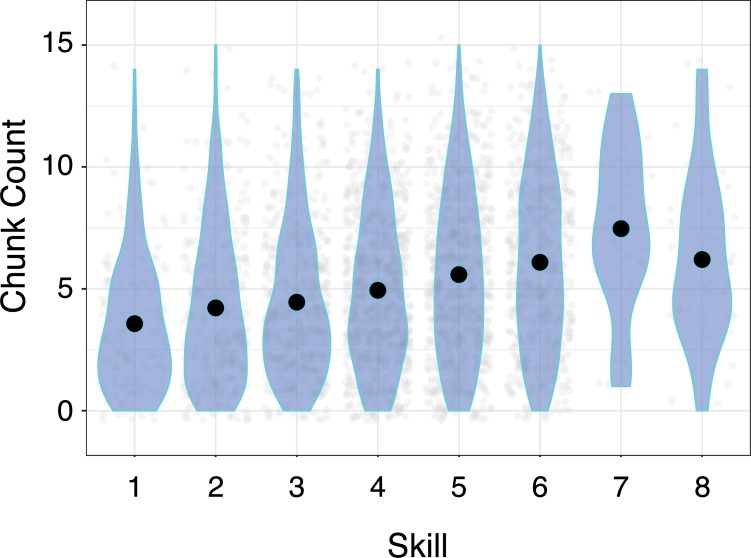
Chunk count by skill. The number of chunks by skill level, where 1 is the least skilled. Each grey point is one game, and the shaded region shows the density of values. The black circle shows the mean value for each skill level.

Our first major finding is that better players rely on a different number of chunks, evidenced by an ANOVA with League and Species to predict the number of critical sequences (F(7, 3178) = 25.87; p<2e-16, η_p_^2^ = 0.054). There is a main effect for the domain-specific nuisance factor player species (F(5,3178) = 51.55; p<2e-16; η_p_^2^ = 0.075). Planned comparisons confirm that more skilled players tend to possess a few more common sequences in their behavioural arsenal (See Fig 3, S1 Table).

**Fig 4:**
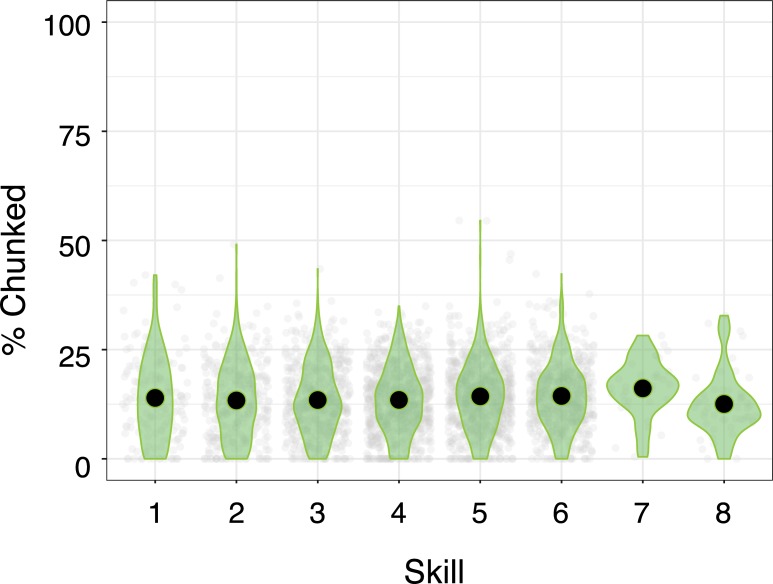
Proportions of actions chunked by skill. The percentage of actions that fall within chunks by skill level.

The second prediction of MCF is that chunks of actions will replace less efficient methods for filling basic game functions, and thus, there will be an overall increase in the proportion of actions falling within chunks. As expected, we identified a main effect of the nuisance factor player species (F(5,3178) = 42.66; p<2e-16, η_p_^2^ = 0.062). However, we found no evidence that proportions of actions falling within chunks changed with skill (F(7,3178) = 1.99; p = 0.053; η_p_^2^ = 0.004). Indeed, chunks appear to take up about 20% of a game’s total actions regardless of skill (Fig 4). As can be seen from planned comparisons reported in S2 Table, the proportion of game actions taken up by common sequences appears to change little, if at all. Our first two findings (an increase in unique chunks, but no change in the proportion of chunked actions) suggest an increase in diversity with skill. This contrasts with the MCF’s prediction of increasingly consistent performance, so we perform followup analyses to probe this predictive failure.

One possible concern with our analysis of prediction 1 and 2 is that they rely on our chunk-detector. However, the MCF predicts that chunk proportions should increase with skill leading to a net decline in the overall variability of behavioural sequences, and it is possible to directly test whether the total number of unique behavioural sequences becomes compressed with skill. If the diversity of behavioural sequences is not reduced with skill, as predicted by the MCFs emphasis on replacing deliberate behaviours with automatic ones, this diminishes the MCF as a viable explanation for expert performance.

One challenge in measuring the diversity of behavioural sequences is that, in the present task-domain, skilled players will trivially have more unique sequences because they produce more actions. Therefore, we sampled 200 actions before and after every game’s ten minute mark, and calculated and summed the number of all unique length 2, 3, and 4 sequences.

StarCraft 2 games vary in duration, and 690 games were dropped for failing to be sufficiently long for 400 actions to be sampled, leaving us with 2501 games for a unique sequencing analysis. The observed number of unique sequences increases with skill. However, a further concern was that a greater number of unique sequences might be explained based on the distribution of action types for a given player (e.g. if players who often employ all of the 7 different types of action will usually have more unique sequences than players who only use 5 types of action). Furthermore, the distribution of action types vary somewhat from player to player and league to league, and so we constructed, for every player, a simulation of the number of unique sequences one would expect given their distribution of action types. This simulation will allow us to see how much motor-sequence diversity should be expected given a players distribution of action types and, consequently, it allows us a method for investigating motor sequence diversity without relying on our chunk-detector.

Our simulation assumed a null distribution where players pick a random permutation of their 400 actions. This is a reasonable facsimile of players choosing actions at random, while maintaining each player's marginal distribution of each actiontype. The goal is to compare the number of unique sequences in random permutations of the actions to the number of unique sequences actually found in the 400 in-game actions. The statistic is the sum of the unique sequences of length 2, length 3, and length 4. For a sample S of 400 actions we call the statistic *S*, and for the original game we call the statistic *G*.

To formally conduct a permutation test, we would need to simulate many different realizations from the null distribution for each player, compute *S* for each realization, and then find the percentile where G falls. Unfortunately, for almost all of the players this percentile is extremely hard to accurately assess because G is almost always much smaller than any corresponding *S*. This is because players aren't picking actions at random. As a surrogate measure for a formal permutation test we construct instead the following statistic: we compare G to the average of 100 samples of S, which we call mean(*S*), and take the difference. This new, derived statistic, randomness, is large (negatively) when the player had few unique sequences in the game, and close to zero if the player picked actions close to randomly.

**Fig 5:**
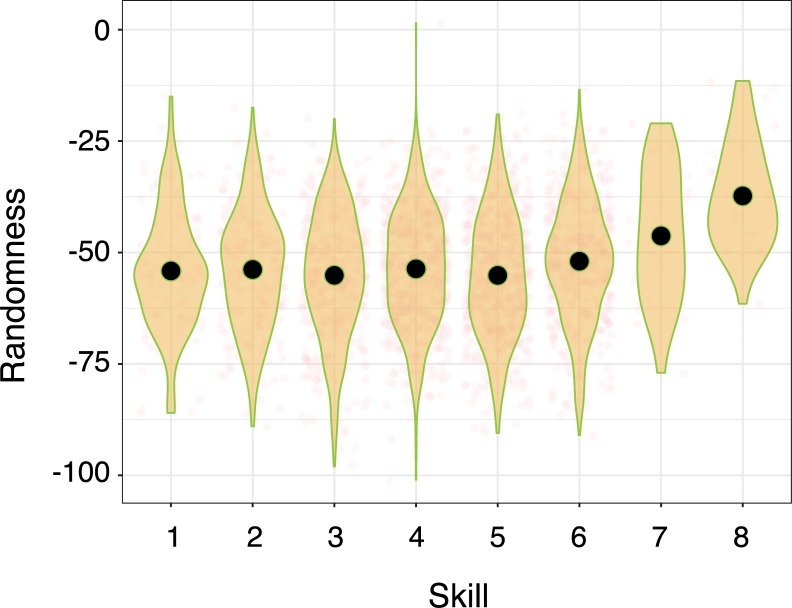
Randomness of sequences by league. Sequence randomness is the number of unique sequences less the number of uniques sequences expected via random sampling. Smaller numbers thus reflect less sequence diversity (and more sequence repetition), and larger numbers reflect more diversity. See the text for further details.

We confirmed the finding of increasing diversity without relying upon chunk detection (see Fig 5), using the difference between G and mean(S) instead. Better players exhibited a greater raw diversity of sequences (F(7,2488) = 10.99, p<2e-16; η_p_^2^ = 0.03) rather than the reduction in diversity predicted by the MCF. Player species was, again, a significant predictor of diversity (F(5,2488) = 235.39; p<2e-16; η_p_^2^ = 0.32). Planned comparisons revealed no significant differences in diversity between the four lowest skill levels, but the higher skill levels exhibited more diverse sequencing behaviour. For example, league 8 differed significantly from skill levels 1–6, and league 6 differed significantly from league 1 (See S3 Table). In short, the MCF fails to explain the increasing diversity of behavioural sequences in this domain. We call this the diversity problem.

### Time saved by chunks

**Fig 6:**
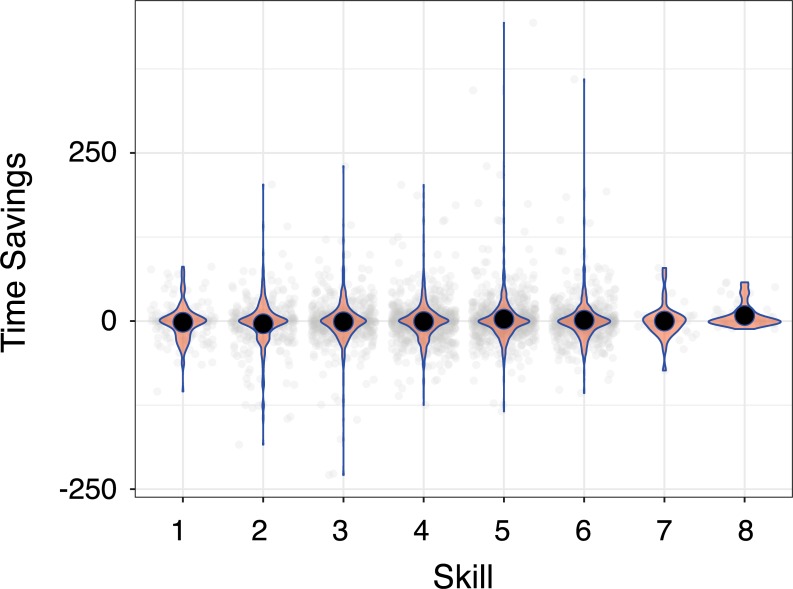
Number of total seconds saved by chunking by league. This is calculated as the average of the duration of chunked sequences less the time for equivalent non-chunked actions for each game by skill level.

**Fig 7:**
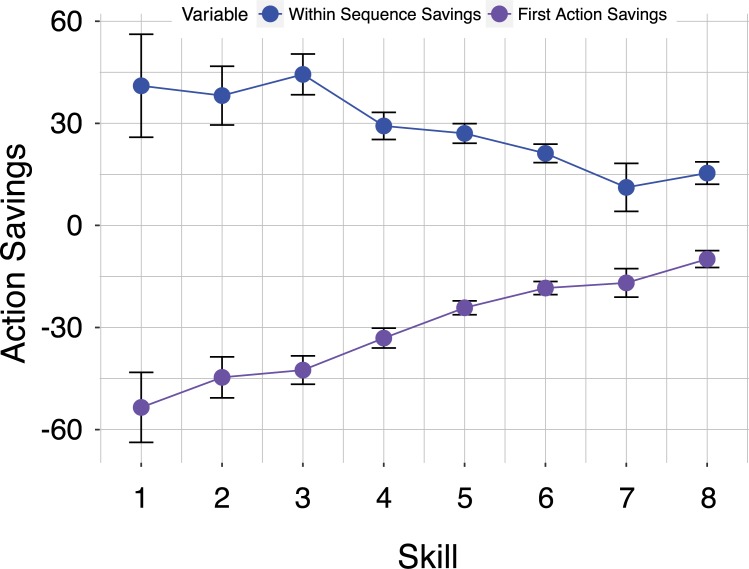
First versus inter-action time savings. Time saved by chunks (in seconds) split for first actions and subsequent actions for each skill level.

In order to measure time savings, we compared chunked action latencies against non-chunked latencies of the same action type. We find no evidence that chunked actions are faster than non-chunked actions (Fig 6; T(3190) = 0.65; p = 0.59; 95% CI = -0.82 : 1.45), and only a weak main effect of skill (F(7,3178) = 2.311, p = 0.02, η_p_^2^ = 0.005) and player species (F(5,3178) = 26.06, p< 2.2e-16, η_p_^2^ = 0.039). The majority of time gained by inter-action latencies appears to be lost in slow first-actions (Fig 7). Furthermore, time savings is very weakly related to average action latency (T(3198) = -8.06; p = 1.05e-16; 95% CI = -0.175 : -0.107, r = -0.14). This implies that as players save more time (i.e., their chunks become faster), raw action latencies tend to get shorter (i.e., actions become faster overall). However, the strength of this relationship is weak, accounting for about 2 percent of the variance in typical action latencies.

Recall that to explain the speed improvements from novices to experts our rough estimates indicated that a chunking explanation requires substantial time-savings for chunks (roughly 1,000ms) and substantial increases in the proportion of a player’s actions that are chunked (~10% more for each level of skill; a 70% increase overall). Less substantial contributions from chunking would mean that chunking must share its explanation of speed gains with other factors.

The present work shows that of the 785ms of skill-based speed improvements that need explaining chunking explains none of it. We found that chunks do not make up nearly enough of the actions to produce the required speed gains (expected, ~80%; obtained, ~20%), nor does the proportion of chunks increase with experience (expected, 10% increase per skill level; obtained, 0% increase per level). We also find no evidence that chunked actions are executed faster than non-chunks (expected, 1000ms savings per chunk; obtained, 0ms savings per chunk).

The clearest description of our data is that players become faster with skill even where (a) sequences are already chunked at the novice levels and (b) even where there is little or no strategic value to the sequence. For example, we considered whether the most frequently used chunks also become even faster with skill. The MCF would predict that these latencies would change the least as they are likely the first skills to reach complete automatization. We examined speeds from the most commonly occurring sequence in our dataset which was Screen-shift—Select: a shift of a players view-screen and the selection of a unit with the mouse. Our chunk detector identified this sequence in 1,789 games. The first action latency for such chunked screen-shifts varied markedly by league (F(7,1776) = 199.7, p<2e-16; η_p_^2^ = 0.44) and, to a much lesser extent, by player species (F(5,1776) = 24.914, p<2e-16; η_p_^2^ = 0.065). The latency of the second action (Select) in these chunks also varied by league (F(7,1776) = 200.13, p<2e-16; η_p_^2^ = 0.44) and player species (F(5,1776) = 5.72, p = 3e-05; η_p_^2^ = 0.015). This shows that while chunks do not appear to save players time, novices nevertheless still have a lot to learn about even the most foundational behavioural sequences in the game.

Interestingly, we also observed learning even in behaviours which are almost irrelevant to success in the game. While the MCF might allow some learning to occur unconsciously and without deliberate attempts to improve, modern accounts of expertise emphasize such deliberate practice in explaining changes in performance [25]. For example, repetitions of right-clicks following screen shifts usually constitute redundant actions in the context of StarCraft 2, as each right-click usually overrides the previous command. We conducted an analysis of this right-click latency without use of our chunk-detector. Instead we identified sequences of right-clicks which contained no other actions and were punctuated between screen shifts. Nevertheless, the average inter-right-click latencies of right-clicks following screen shifts changed across the first six leagues of experience (F(5,3196) = 79.71, p<2e-16; η_p_^2^ = 0.11; but see S1 Text for subtle respects in which this analysis departs from other analyses presented here). As one would expect from redundant actions that are common to all game species, there was no main effect for player species (F(2,3196) = 2.62, p = 0.07, η_p_^2^ = 0.001).

**Fig 8:**
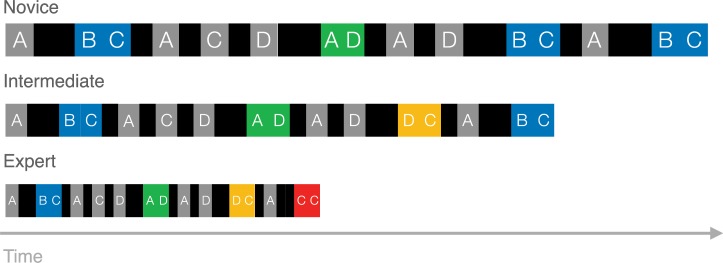
Schematic illustration of how action sequences change with expertise in our data. As skill is acquired, speed is gained through a general speedup of all actions.

The overall pattern of results, therefore, is characterized not by the chunking account illustrated in Fig 1, or even the effects of deliberate practice alone, but by the illustration shown in Fig 8. The proportion of actions that are chunked does not change with increased learning, however, the number of different chunks does increase as players gain experience. Overall, experts show increasing diversity in their actions, rather than increasing constancy predicted by the MCF, which we call the diversity problem. Finally, first action latencies are longer than non-chunked actions, eliminating the benefit of decreased latencies for subsequent actions in chunks. Learning-related speed increases, it seems, come not as a result of chunking, but largely from a general speed increase to all atomic actions, called here the time savings problem.

## Discussion

The Motor Chunking Framework claims that chunks save time: actions are performed in a learned sequence and so do not require individual planning, thus, actions after the initial action are executed faster. As illustrated in Fig 1, the MCF explanation for expert performance involves the conversion of an increasing proportion of individual actions into timesaving chunks. We sought to test these predictions about the proportion, diversity, and speed of chunks using replay data from the real-time strategy game StarCraft 2, collected from players across eight skill levels.

Overall, the findings of the present work stand against, rather than for, the MCF as an explanation of skill development in StarCraft 2; performance, in turns out, is not merely a story about the monopolizing influence of a relatively small number of highly adaptive behavioural sequences or chunks. The MCF account has two problems: a time-saving problem, and a diversity problem.

The time-saving problem stems from the simple fact that, in our data, chunks do not save players time overall. This, alone, undermines chunking as an explanation for the clear and consistent trend of faster performance as skill develops. But there is an additional and related difficulty—the percentage of actions that are in chunks does not increase with expertise, so a uniform effect of chunking cannot account for skill related changes. Even if every chunked action did save some set amount of time, the MCF is thus still unable to account for skill-based performance gains.

The MCF is also at odds with the overall trend of increasing diversity of actions as skill increases. If useful chunks were replacing non-optimal non-chunked sequences, then we would see an increase in the number and variety of chunks, but an overall decrease in behavioural diversity—random or accidental actions would drop away, leaving only optimal, automatized chunks. We find evidence to the contrary: the proportion of actions that are chunked remains constant as skill develops, such that the few additional chunks that are gained with expertise lead to increasing, rather than decreasing, action diversity.

### Methodology-based alternative explanations for our findings

We acknowledge that complicated methodologies can impede the interpretation of findings. Unfamiliarity with the domain, or the methods used might justifiably make one uneasy about the striking findings in the present work. In this section we will address the most common concerns.

*Are our results due to factors specific to StarCraft 2*? We find it difficult to resolve the Diversity Problem and the Time Savings Problem by appealing solely to domain specific idiosyncrasies. It is not very plausible that some poorly understood game mechanic of StarCraft 2 prevents common sequences from monopolizing more than 20% of game actions. Actions of type ‘Train’ alone typically occupy 10% or more of the overall game actions and these appear ripe for the automatization. Training always requires either a ‘Select’ or a ‘Hotkey’ command to select relevant production structures prior to training, and funds limit players from training all their required units once. Instead, the production of a powerful army typically requires that players diligently task switch from other objectives back to issues of army production. Automatizing such sequences would presumably be faster, require less cognitive load, and have no obvious drawbacks. The same considerations apply to other typical StarCraft tasks such as expanding and positioning one’s army. StarCraft 2 is a domain where we should expect the MCF to thrive.

*These data show that even novices have chunks*, *is that an indication that the chunk detector is faulty*? Bronze league players, the weakest players in our sample, report an average of 264 hours of practice and are therefore much more skilled than the novices in typical expertise research. We should expect that their play will already contain some automatized skills. Indeed, even the least experienced person in the dataset, with only 12 hours of reported experience, has plenty of time to develop *some* motor chunks.

*These data show that some players have no chunks*, *is that an indication that the chunk detector is faulty*? While we believe that players have sufficient experience to develop motor chunks, we also are mindful that StarCraft 2 is a new domain, and quite different from laboratory tasks in many ways. In laboratory studies of motor chunking the participants participants do not have to figure out what sequences are effective, they are simply provided them. Participants are typically producing only a few sequences and are repeating them often. Finally, in the lab participants are in situations of low distraction. In StarCraft 2 players have none of these advantages, and so we do not find it difficult to believe that chunk acquisition might be considerably slower in StarCraft 2 than in the laboratory, and that a few players might be unusually slow to automatize. We note that the overwhelming majority of participants (94%) do indeed develop chunks. It is also important to note that, given that our chunk analyses is based on a sample of over 800,000 chunks, a small number of missed chunks is very unlikely to influence our findings.

*Could a faulty chunk detector be the cause of our findings*? Any chunk-detection algorithm will occasionally miss chunks or produce false alarms. In the case of our chunk-detector, skilled players might have chunked complex maneuvers which are only required once per game, so our algorithm will never categorize these behaviours as chunked. It is especially important, therefore, to consider how limitations in the chunk-detector could impact our analysis.

First, the chunks that were identified by our detector show the standard pattern [2, 3] of slow first actions (first actions average, 720ms while subsequent actions are 563ms). If there are false alarms, there are not so many as to obscure the expected slowness of first actions. For this reason it seems unlikely that we have a large number of misclassifications, generally.

An alternate possible concern is that our chunk detector is only missing real chunks rather than misclassifying everything. This would mean that when we calculate time saved by chunks we are comparing chunk latencies, to a mixture of non-chunk and missed chunk latencies. If we missed only a few chunks, then the observed effect of time saved by chunks would be smaller than it should be because the missed chunks would bringing the mean of the non-chunk (in reality a mixture) group closer to the mean of the chunk group. If the mixture group contains almost all missed chunks, exhibiting a high miss rate, then the time savings would be near zero. There are thus two factors to keep in mind. The first factor is the true difference in latencies between chunks and non-chunks (i.e., the per-action time savings), and the second is how many chunks might be reasonably missed.

Missed chunks are not a convincing explanation of our findings because there are no plausible values for per-action time savings of chunked actions, or for the percentage of chunks, that are consistent with the MCF’s account of action latencies. The problem is that per-action time savings must be large for chunks to account for the significant speed-up of actions with expertise—recall, latencies of the lowest skill participants are about 4 times slower than than of the highest skill participants (a difference of about 700ms; see Fig 2). The second factor is the proportion of the actions labeled non-chunks that are actually missed chunks. To make a large (population) difference between chunked latencies and non-chunked latencies evaporate, this proportion must also be very large (if this proportion is small then missed chunks might reduce time savings, but it would not be sufficient to eliminate them). Due to the size of our sample, we have about a 99% chance to detect 3.5 seconds of overall time savings per game, so it would need to be the case that most actions are chunked and that most chunks are missed. But, even that scenario is problematic, because if most actions are already chunked, then how do we explain the novice to expert speed increase? The only way such an explanation is possible is if our chunk detector is very good at capturing novice chunks but systematically misclassifies the vast majority of all of the expert actions as non-chunked. Unfortunately, while it is plausible that the assumed relationship between sequence prevalence and chunkhood becomes weaker in experts (who might practice rare sequences outside of real games), it seems unlikely that this behaviour could lead to the extreme bias which would be required to make our results fit with the MCF. After all, we observe latency shrinkage even for redundant actions like right-clicks that professionals have no reason to practice.

Finally, it is important to reiterate that missed chunks cannot explain away the diversity problem at all. We verified the original finding using a second, independent analysis which makes no reference to our chunk detector by sampling 200 actions before and after the ten minute mark of a game (Fig 5). Missed chunks, for the reasons described above, are not a viable alternative account.

*Does the analysis still hold for nested chunks*? A final possibility worth considering whether our results could be influenced by the fact that larger chunks are often *nested* within smaller ones [4]. There is a sense in which our analysis overlooks these superchunks, as our chunk-detector only identifies possible chunks up to four actions in length. However, our time-savings analysis does consider nested chunks in the sense that, after identifying lower-level chunks, we flag every action within the game as being chunked or not. The first action of a chunked sequence is defined as the first action in a sequence of chunked actions. Therefore, while our chunk-detector only identifies two, three, and four action chunks, our analysis does give appropriate consideration of nested chunks. Indeed, the time savings described in Fig 7 is meant to represent nested chunks of any size.

How do these results fit with previous attempts to identify chunks in StarCraft 2?

Thompson, McColeman, Stepanova, & Blair [19] defined chunks as any action sequence falling between screen-shifts. They also found the standard latency pattern of slow first actions [2, 3]. The two chunk-detection algorithms may overlap, as the most common chunk was ‘Screen-shift—Select’, suggesting that attentional shifts may indeed be reasonable indicators of a chunk-boundary.

### Concluding remarks

The MCF predicts a net decrease in the diversity of expert sequences. Our unique sequence analysis is a direct examination of this prediction finding that, contrary to MCF, the variety of behavioural sequences executed by better players is closer to a random shuffling of their action types. Diversity might be adaptive, much like expert drivers that display a more diverse fixation pattern than novices while operating a car [32]. Underwood and colleagues suggest that their observation of more random fixation patters in better drivers is a consequence of reduced cognitive load during the primary driving task. It may be the case that expert StarCraft 2 players are exhibiting less cognitive load during their primary interfacing task and dedicated more cognitive resources to higher level and exploratory processes. If this account is correct, then the role of motor chunking is about freeing cognitive resources, not saving time directly through automatization.

The Time Savings problem is another challenge to the MCF. The discrepancy between time savings of first actions savings and within action savings suggests that our detector was indeed picking out hierarchically controlled sequences. However, on standard theories of automatization, speed and automatization are purchased by experience [5] and there is every reason to think that StarCraft 2 players have more experience executing prevalent sequences than less prevalent sequences (recall that our chunk detector highlights sequences that are highly frequent relative to the base rates of their components). Nevertheless, we struggled to find any evidence that chunks save time.

Actions of better StarCraft 2 players are much faster, and if these speed differences are not purchased by increased automatization, then they must presumably be purchased by the selection of better chunks. In other words, it may be that skill development in StarCraft 2 is less of a story about automatization and more of a story about maximal adaptation to task constraints [33]. In this proposal, better players are those who have identified the most efficient ways of accomplishing basic game functions. There is also some evidence that experts opt for more efficient forms of commanding their army. For example, players can select units either with natural ‘Select’ commands or more efficiently with ‘hotkey’ commands, and there is evidence that better players make better use of interface shortcuts [34].

One difficulty with explaining expert speed by looking to the quality of expert behavioural sequences is that this does not solve the diversity problem. Experts, as a class, do not appear to settle upon a shared way of doing things. On the contrary, there appears to be respectable variability across all players in terms of the actual chunks they employ. Recall that, of the unique critical sequences, a sequence was usually only critical in about four games. Amongst professional players, the median number of games in which each critical sequence was statistically significant was two.

One might try to resolve both the Diversity Problem and the Time Savings problem by arguing that (a) increased diversity in behavioural sequences are due to a reduction in cognitive load, allowing for a host of exploratory behaviours and (b) the sequences of StarCraft 2 players are all too automatized for well practiced sequences to save time. For this proposal to be tenable, automaticity is relegated to the position of a necessary criterion for competent performance with no capacity to explain between-subject differences in speed (i.e., the speed differences in Fig 2). While the explanatory role of automaticity in expert performance has been questioned previously [33], it has never, to our knowledge, sunk quite so low in a major theory of skill development.

It is important to note that the diversity problem and the time-savings problems, while perhaps a serious threat to a simple theory of motor chunking, are actually opportunities for more nuanced theories of chunking. For example, a motivation for the Cognitive framework for Sequential Motor Behaviour (C-SMB; [21]) was to ‘[…] to increase the awareness of cognitive scientists and cognitive neuroscientists of the processing complexities involved in preparing and executing even relatively simple motor sequences.’ ([21], p. 56). Motor chunks, on this account, are mental representations available to response selection processes, and there are a variety of motor execution strategies that may make more or less use of motor chunks.

This sort of theory might be able to accommodate our results in a number of ways. It might address the diversity problem by positing that the cognitive systems of novice StarCraft 2 players, being adaptable, restrict behaviour to the relatively small repertoire of chunks that were mastered within the first 100 hours of practice. This could resolve some aspects of the diversity problem, as it would explain why action sequences become more diverse with skill. Other aspects of the diversity problem, such as the consistent proportions of chunked actions, remain somewhat puzzling as the mechanics of StarCraft 2 would seem to provide for many avenues for chunking.

The time savings problem, while certainly counter-intuitive, is not necessarily a problem for modern chunking theories. The C-SMB, for example, is reconcilable with the notion that motor-chunks play no direct and major role in explaining skilled performance timings beyond a few hours of practice. The C-SMB posits a variety of different chunk-types and execution strategies. The important explanatory role of motor chunks might be exhausted after the first few hours of practice.

Consequently, we are not arguing that chunks don’t exist. On the contrary, the chunks we detected did show the anticipated delayed first actions. What our findings suggest is that simple motor chunking is an inadequate explanation of the timings and diversity of learned motor sequences in complex tasks. Given that during extended practice, there are many documented structural [35–39], functional [40] and connectivity changes in the human brain [41] that can potentially explain performance changes without reference to chunking, perhaps it is time to reevaluate the central role chunking has played in our understanding of skilled performance. At the very least, our work echoes the call for more developed and nuanced accounts of motor-chunking (e,g. [21]), as we find no evidence that uncovering the neurobiological implementation of chunking in the motor system will yield a full or significant account of expert behaviour.

In short, it appears that the motor chunking framework’s straightforward explanation of StarCraft 2 performance is flawed in a fundamental respect. This was a domain where we would have thought that the MCF would successfully predict performance since speed is critical for success and there are a limited set of possible actions. However, instead of a simple story where automatization leads a small set of highly efficient and automatic sequences to take over the functions of basic StarCraft 2 mechanics, we find a greater diversity of sequences with highly skilled individuals, and little evidence that players are faster when executing the most common sequences. Without some solution to the Diversity problem and the Time Savings problem, it appears that MCF will have little application to the study of expertise beyond the domains of traditional study such as typing.

## Supporting information

S1 Text Supplementary information. (DOCX)

S2 Text Dictionary of columns used in S1 File–S5 File. (PDF)

S1 Fig Sample size by skill level. (EPS)

S1 Table Number of critical sequences by skill. The number of critical sequences by skill (t values are adjusted for family wise error).
(EPS)

S2 Table Proportion of actions in a CS by skill. Proportion of actions in a CS by skill (t values are adjusted for family wise error).
(EPS)

S3 Table Simulated-Observed Unique Sequence Count by skill. Simulated-Observed Unique Sequence Count by skill (t values are adjusted for family wise error).
(EPS)

S4 Table Time savings by skill. Time savings by skill (t values are adjusted for family wise error).
(EPS)

S1 File GameLevel. (CSV)

S2 File FirstActionVSOtherAction. (CSV)

S3 File RightClickSpeed. (CSV)

S4 File UniqueSequences. (CSV)

S5 File Screen_Sel. (CSV)
