# Supplementary material for: Classic motor chunking theory fails to account for behavioural diversity and speed in a complex naturalistic task
Source: PLoS One. 2019 Jun 13;14(6):e0218251. doi: 10.1371/journal.pone.0218251 (PMC6564011; doi:10.1371/journal.pone.0218251)
Supplement: S2 Text — (PDF) [file pone.0218251.s002.pdf]

Primary measures

Reported measure	Table.variableName	Description
**LeagueIdx**	S1_File: LeagueIdx<br>S3_File: LeagueIdx<br>S4_File: LeagueIdx<br>S5_File: LeagueIdx	Leagues are ordinal categories indicating skill level. One is the lowest and eight is the highest.
**RawSpeed**	S1_File: RawSpeed	The average latency (ms) of all action types in the game, regardless of whether they are chunked or not
**Latency (First vs inter-action)**	File_S2: Latency	Mean latencies (s), divided up by whether they reflect the first actions in a chunked sequence (cscoding=2) or inter-action latencies (cscoding=1).
**Number of critical sequences**	File_S1: CriticalSequenceCount	How many sequences are identified by our chunk detector as critical sequences
**Proportion of actions in chunks**	File S1: ProportionCSAction	The proportion of actions that within common sequences/ chunks
**TotalTimeSavings**	File_S1: TotalTimeSavings	The latency (s) of actions made in chunked sequences/chunks versus the average latency of the same actions made outside of chunked sequences.
**Randomness**	File_S4: Randomness	The difference between the unique sequences observed in a sample of 400 actions from a player less the same actions in a random order. Lower values mean less randomness, values approaching 0 mean more randomness.
**Screen shift times**	S5_File: ps_times	Mean latency (s) for screen-shifts in the common chunked sequence 'Screen shift-Select'
**Select times**	S5_File: Sel_Times	Mean latency (s) for select commands in the common chunked sequence 'Screen shift-Select'

Reported measure	Table.variableName	Description
**RCLatency**	S3_File: RCLatency	Actions are first segmented into those falling within Screen Shifts and those falling outside of Screen shifts (see supplementary materials for details). Screen Shifts containing only Right-Click commands have the inter-action latency means calculated. A player's RCLatency (ms) is the mean of their inter-action latency means.

All Columns by Table

S1 File: GameLevel.csv

Column	Plain language	Description
**GameID**	Game number identifier	The identifier for the game. There is one unique value per game in the dataset.
**LeagueIdx**	League number	Leagues are ordinal categories indicating skill level. One is the lowest and eight is the highest.
**Race**	Game species.	Players can control three different kinds of armies. “Race” is a term in StarCraft that indicates which type of army the player controls.
**CriticalSequenceCount**	Number of critical sequences	How many sequences are identified by our chunk detector as critical sequences.
**ProportionCSAction**	Proportion of actions in chunks	The proportion of actions that within common sequences/ chunks.
**TotalTimeSavings**	The temporal advantage of chunked actions	The latency (s) of actions made in critical sequences/chunks versus the latency of actions made outside of critical sequences.
**RawSpeed**	Average action latency	The average latency (ms) of all action types in the game, regardless of whether they are chunked or not

S2 File: FirstActionVSOtherAction.csv

Column	Plain language	Description
**GameID**	Game number identifier	The identifier for the game. There is one unique value per game in the dataset.
**CSCoding**	Critical sequence property	Aggregate data for chunked sequences. Values of 1 reflect inter-action latencies (actions that are preceded by chunked actions) and values of 2 refer to actions which begin a sequence of chunked actions.
**Latency**	Time to take an action	Refers to the mean action latencies (seconds)

S3 File: RightClickSpeed.csv

Column	Plain language	Description
**GameID**	Game number identifier	The identifier for the game. There is one unique value per game in the dataset.
**LeagueIdx**	League number	Leagues are ordinal categories indicating skill level. One is the lowest and eight is the highest.
**RCLatency**	The time it takes a player to enact repeated Right-Clicks in a row.	Actions are first segmented into those falling within Screen Shifts and those falling outside of Screen shifts (see supplementary materials for details). Screen Shifts containing only Right-Click commands have the inter-action latency means calculated. A player's RCLatency (ms) is the mean of their inter-action latency means.

S4 File: UniqueSequences.csv

Column	Plain language	Description
**GameID**	Game number identifier	The identifier for the game. There is one unique value per game in the dataset.
**Race**	Game species.	Players can control three different kinds of armies. “Race” is a term in StarCraft that indicates which type of army the player controls.
**LeagueIdx**	League number.	Leagues are ordinal categories indicating skill level. One is the lowest and eight is the highest.
**NumberOfUniqueSequences**	Number of distinct sequences.	The number of unique length 2, 3, and 4 sequences found in 200 actions before and after the ten-minute mark of a game. Null values reflect games that did not have 200 actions before and after the ten minute mark.
**Simulated**	Simulated sequences	The simulated number of length 2, 3, and 4 sequences found by randomly permuting the 200 actions before and after the ten-minute mark of a game. Data are based on the average of 100 simulations.
**Randomness**	Diversity of sequences	The difference between Simulated and NumberOfUniqueSequences

S5 File: Screen_Sel.CSV

Column	Plain language	Description
**GameID**	Game number identifier	The identifier for the game. There is one unique value per game in the dataset.
**Race**	Game species.	Players can control three different kinds of armies. “Race” is a term in StarCraft that indicates which type of army the player controls.
**LeagueIdx**	League number.	Leagues are ordinal categories indicating skill level. One is the lowest and eight is the highest.
**Screen_Shift_Times**	Latency for screen-shifts in the common chunked sequence ‘Screen shift-Select'	Mean latency (seconds) for screen-shifts in the common chunked sequence ‘Screen shift-Select'
**Sel_Times**	Latency for select commands in the common chunked sequence ‘Screen shift-Select'	Mean latency (seconds) for select commands in the common chunked sequence ‘Screen shift-Select'
